# Integrated multi-omics reveals acylcarnitine accumulation as an early driver of doxorubicin-induced cardiotoxicity targeted by chlorogenic acid

**DOI:** 10.3389/fphar.2026.1865654

**Published:** 2026-07-30

**Authors:** Jing Ren, Yibo Yang, Guangdong Li, Huiying Sha, Bing Wang, Yin Wang, Ning Cui, Taoyan Liu

**Affiliations:** 1 Department of Respiratory and Critical Care Medicine, The Affiliated Hospital of Qingdao University, Qingdao, Shandong, China; 2 Institute for Translational Medicine, College of Medicine, The Affiliated Hospital of Qingdao University, Qingdao University, Qingdao, China; 3 Biomedical Center of Qingdao University, Qingdao, China

**Keywords:** acylcarnitines, cardiotoxicity, chlorogenic acid, doxorubicin, lipidomics, PPAR

## Abstract

**Introduction:**

Doxorubicin (DOX)-induced cardiotoxicity (DIC) is a major clinical challenge, with early metabolic reprogramming acting as a potential pathogenic driver. This study investigates the early lipid metabolic disturbances in DIC and evaluates the cardioprotective potential of the natural metabolic modulator, chlorogenic acid (CGA).

**Methods:**

Transcriptomic analysis was utilized to identify initial metabolic alterations. Lipidomics and spatial metabolomics were performed to map lipid distributions in DOX-treated H9c2 cardiomyocytes and C57BL/6J mouse hearts. The therapeutic mechanisms of CGA were assessed *in vivo* through echocardiography and histopathology, and *in vitro* using pharmacological interventions targeting the PPAR-FAO axis.

**Results:**

Transcriptomics and lipidomics revealed that DOX significantly suppresses the PPAR signaling pathway and fatty acid oxidation (FAO), resulting in the accumulation of ceramides and long-chain acylcarnitines. Spatial metabolomics confirmed *in situ* myocardial acylcarnitine accumulation. CGA treatment effectively improved cardiac function, mitigated oxidative stress, and inhibited apoptosis. Mechanistically, CGA reduced mitochondrial ROS and acylcarnitine levels by restoring PPAR expression and promoting FAO. Pharmacological blockade of PPARα or exogenous addition of acylcarnitines completely abolished CGA’s protective effects.

**Discussion:**

Impaired FAO and the subsequent accumulation of toxic acylcarnitines serve as early drivers of DOX-induced metabolic dysfunction. Targeting this lipid metabolic dysregulation with CGA represents a promising therapeutic strategy for mitigating anthracycline-induced cardiotoxicity.

## 1 Introduction

Doxorubicin (DOX) is one of the most effective and widely used anthracycline chemotherapeutic agents for the treatment of both hematological and solid malignancies. However, its clinical application is substantially limited by cumulative and often irreversible cardiotoxicity, which can progress to heart failure long after cancer treatment has ended. Traditionally, oxidative stress has been considered the primary driver of DOX-induced cardiotoxicity. Nevertheless, the failure of antioxidant-based therapies to provide meaningful clinical protection has challenged this canonical view, pointing instead to alternative mechanisms, including mitochondrial dysfunction, DNA damage, and impaired iron handling (Lin et al., 2025; Dulf et al., 2024; Liu et al., 2020). Among these, mitochondrial dysfunction is regarded as a central pathogenic event because it directly induces metabolic reprogramming, shifting cardiac energy metabolism toward an inefficient, embryonic-like state (Abdullah et al., 2022). Emerging evidence further suggests that early metabolic reprogramming is not merely a downstream consequence of mitochondrial injury but may actively contribute to disease initiation through the accumulation of bioactive metabolic intermediates and epigenetic remodeling (Chen et al., 2025; Arif et al., 2024). Because metabolic alterations occur rapidly and often precede transcriptional adaptations, they may represent upstream drivers of cardiotoxicity, highlighting the urgent need to identify therapeutic strategies that specifically target these early metabolic disturbances.

The adult heart depends primarily on fatty acid oxidation (FAO) for ATP production, making efficient lipid metabolism critical for cardiac function (Dong et al., 2021; Mendez and Ortiz, 2021). In doxorubicin-induced cardiotoxicity (DIC), impaired mitochondrial FAO is a consistent metabolic abnormality that results in reduced energy production and the accumulation of incompletely oxidized fatty acid metabolites (Mitra et al., 2008; A et al., 1997). Among these metabolites, acylcarnitines have emerged as sensitive indicators of mitochondrial FAO dysfunction and are increasingly implicated in the pathogenesis of cardiovascular diseases. Excessive acylcarnitine accumulation can disrupt mitochondrial homeostasis, promote oxidative stress, impair cellular signaling, and induce cardiomyocyte injury. Because DOX suppresses PPAR-regulated fatty acid metabolic pathways, acylcarnitine buildup may represent a direct metabolic consequence of FAO failure (Renu et al., 2022; Liu et al., 2025). However, whether acylcarnitine accumulation is simply a marker of metabolic dysfunction or an active contributor to DIC development remains largely unknown. Clarifying this relationship may identify novel metabolic targets for cardioprotection during DOX chemotherapy.

Chlorogenic acid (CGA) is a polyphenolic compound widely found in plants such as Eucommia ulmoides, *Lonicera japonica* (honeysuckle), coffee, potatoes, apples, and tea leaves (Lu et al., 2020), and it exhibits good safety and tolerability. As a natural bioactive molecule, CGA has demonstrated significant anti-inflammatory, antioxidant, lipid-lowering, glucose-lowering, and immunomodulatory effects in various chronic metabolic and age-related diseases (Naveed et al., 2018; Wu et al., 2020; Pimpley et al., 2020), indicating its broad therapeutic potential. In models of DOX-induced cardiotoxicity, CGA exerts anti-apoptotic and cardioprotective effects by activating the Nrf2/HO-1 signaling pathway and regulating kynurenine metabolism (Cicek et al., 2023). A growing body of evidence further indicates that CGA plays a central role in metabolic regulation, particularly in lipid metabolism. CGA activates AMPK, upregulates CPT1, and inhibits ACC, thereby promoting fatty acid β-oxidation and reducing triglyceride and free fatty acid levels in the liver and plasma. At the same time, it suppresses key lipid-metabolizing enzymes involved in fatty acid synthesis and cholesterol esterification, improving obesity and glucose metabolic disorders. In hepatocytes and chronic endotoxin exposure models, CGA markedly reduces lipid droplet accumulation, restores lipid homeostasis, and lowers transaminase levels (Yan et al., 2022). Moreover, by enhancing the supply of acetyl-CoA derived from fatty acid β-oxidation, CGA promotes stem cell proliferation and lipid biosynthesis (Zong et al., 2025).

Based on these findings, this study aims to elucidate the early initiating patterns of lipid metabolic dysregulation during DOX-induced myocardial injury, to identify the key lipid metabolic alterations that drive cardiomyocyte damage, and to evaluate whether CGA—with its metabolic regulatory properties—can alleviate DOX-induced cardiotoxicity by targeting and correcting the above lipid metabolic disturbances.

## 2 Materials and methods

### 2.1 Reagents and antibodies

Doxorubicin (D107159) was procured from Aladdin (Shanghai, China). Chlorogenic acid (PS0131-1000) was obtained from Push Bio-technology (Chengdu, China). DHB was obtained from ProteoChem (Hurricane, UT, United States), trifluoroacetic acid (TFA) were purchased from Sigma-Aldrich (St. Louis, MO, United States). LC-MS grade water and acetone were purchased from Fisher Scientific (Fair Lawn, NJ, United States). Primary antibodies against PPARγ, PPARα, PPARβ and 4-hydroxynonenal were purchased from Santa Cruz (Dallas, Texas, United States). Antibodies against Bax, Bcl-2, cleaved caspase 3 were purchased from HuaBio (Hangzhou, China). GAPDH antibody were purchased from Santa Cruz Biotechnology (Dallas, Texas, United States). Secondary antibodies Alexa Fluor 488 Goat anti-Mouse IgG (H + L) and Alexa Fluor 568 Goat anti-Rabbit IgG (H + L) were purchased from ZSGB-BIO (Beijing, China). 4′,6-Diamidino-2-phenylindole (DAPI) was purchased from Beyotime Biotechnology (Shanghai, China). The Free Carnitine and Total Carnitine Content Assay Kit (BC0675) and the Fatty Acid Oxidation (FAO) Rate Assay Kit (BC0815) were purchased from Solarbio Science and Technology (Beijing, China).

### 2.2 Animal model

All animal experiment procedures were approved by the Institutional Animal Care and Use Committee of Qingdao University (Approval NO.: QDU-AEC-2024072). Male C57BL/6J mice were obtained from GemPharmatech (Shanghai, China) and maintained under standard conditions (24 °C ± 1 °C, 12 h light/dark cycle) with free access to food and water. Mice were randomly divided into four groups (n = 8 per group) as follows: Control group: received saline daily for 14 days; CGA group: received chlorogenic acid (100 mg/kg/day, oral gavage) for 14 days and a single intraperitoneal injection of saline on day 10; Dox group: received a single intraperitoneal injection of doxorubicin (15 mg/kg) on day 10; and DOX + CGA group: received chlorogenic acid (100 mg/kg/day, oral gavage) for 14 days and a single intraperitoneal injection of doxorubicin (15 mg/kg) on day 10.

### 2.3 Cell culture

H9c2 cardiomyoblasts were obtained from the Cell Bank of the Chinese Academy of Sciences (Shanghai, China). Cells were cultured in high-glucose DMEM (Servicebio, Wuhan, China) supplemented with 10% (v/v) fetal bovine serum (Lonsera, Hangzhou, China) and 1% (v/v) penicillin–streptomycin (10,000 U/mL penicillin and 10 mg/mL streptomycin; Servicebio). Cells were maintained at 37 °C in a humidified atmosphere containing 5% CO_2_, and the medium was replaced every 2 days. Cells were treated with 5 μM doxorubicin alone or in combination with chlorogenic acid as indicated.

### 2.4 Cell viability detection

Cell viability was assessed using a CCK-8 assay kit (Lancosa, Jinan, China) according to the manufacturer’s instructions. Briefly, cells were seeded in 96-well plates at a density of 5 × 10^3^ cells per well and incubated for 24 h. After treatment with doxorubicin for 24 h, CCK-8 solution was added to each well, and absorbance at 450 nm was measured using a microplate reader.

### 2.5 Biochemical analysis

ABiochemical parameters were measured using a Chemray 800 automatic analyzer (Rayto, Shenzhen, China). Commercial assay kits (Coibobio, Shanghai, China) were used to determine lactate dehydrogenase (LDH), creatine kinase (CK), and creatine kinase-MB (CK-MB) levels according to the manufacturer’s protocols.

### 2.6 Mitochondrial membrane potential assay

Mitochondrial membrane potential (ΔΨm) was assessed using a JC-1 assay kit (Beyotime Biotech, Shanghai, China). H9c2 cells were seeded in six-well plates and incubated with JC-1 working solution for 20 min at 37 °C. Fluorescence signals were detected using a fluorescence microscope according to the manufacturer’s instructions.

### 2.7 Detection of mitochondrial ROS and cell ROS

H9c2 cells were seeded in six-well plates and incubated with 150 nM MitoSOX or CellROX dye (Beyotime Biotech, Shanghai, China) for 15 min at 37 °C. Cells were then washed three times with PBS and stained with Hoechst (1 μg/mL) for 25 min. After washing, fluorescence images were captured using a confocal fluorescence microscope (Leica, Wetzlar, Germany).

### 2.8 Echocardiogram evaluation

Cardiac function was evaluated using a Vevo 3100LT high-resolution small animal ultrasound imaging system (VINNO Technology, Suzhou, China). Mice were anesthetized and placed in a supine position. Parameters including left ventricular internal diameter in systole (LVIDs), left ventricular internal diameter in diastole (LVIDd), left ventricular ejection fraction (LVEF), and left ventricular fractional shortening (LVFS) were measured.

### 2.9 RNA extraction and real-time quantitative PCR

Total RNA was extracted from tissues using TRIzol reagent (Invitrogen, Carlsbad, CA, United States) according to the manufacturer’s instructions. RNA concentration and purity were determined prior to reverse transcription. For quantitative real-time PCR (qRT-PCR), 0.1 μg of total RNA was used with a one-step RT-PCR kit (Sangon Biotech, Shanghai, China). Gene-specific primers were designed for target genes. GAPDH was used as an internal control. Primer sequences are listed below:

**Table:** 

Gene	​	Sequence
*Pparg*	Forward primer	5′-GTA​CTG​TCG​GTT​TCA​GAA​GTG​CC-3′
​	Reverse primer	5′-ATC​TCC​GCC​AAC​AGC​TTC​TCC​T-3′
*Ppara*	Forward primer	5′-ACC​ACT​ACG​GAG​TTC​ACG​CAT​G-3′
​	Reverse primer	5′-GAA​TCT​TGC​AGC​TCC​GAT​CAC​AC-3′
*Ppard*	Forward primer	5′-GGA​CCA​GAA​CAC​ACG​CTT​CCT​T-3′
​	Reverse primer	5′-CCG​ACA​TTC​CAT​GTT​GAG​GCT​G-3′
*Hcar2*	Forward primer	5′-CTG​TTT​CCA​CCT​CAA​GTC​CTG​G-3′
​	Reverse primer	5′-CAT​AGT​TGT​CCG​TCA​GGA​ACG​G-3′
*Mgll*	Forward primer	5′-GAC​ACC​ATC​CAG​AAG​GAC​TAC​C-3′
​	Reverse primer	5′-GAT​TGG​CAA​GGA​CCA​GAG​GTG​A-3′
*Hmgcr*	Forward primer	5′-GCT​CGT​CTA​CAG​AAA​CTC​CAC​G-3′
​	Reverse primer	5′-GCT​TCA​GCA​GTG​CTT​TCT​CCG​T-3′
*Alox5*	Forward primer	5′-TCT​TCC​TGG​CAC​GAC​TTT​GCT​G-3′
​	Reverse primer	5′-GCA​GCC​ATT​CAG​GAA​CTG​GTA​G-3′
*Tlr4*	Forward primer	5′-AGC​TTC​TCC​AAT​TTT​TCA​GAA​CTT​C-3′
​	Reverse primer	5′-TGA​GAG​GTG​GTG​TAA​GCC​ATG​C-3′
*Hdac1*	Forward primer	5′-TGA​AGC​CTC​ACC​GAA​TCC​GCA​T-3′
​	Reverse primer	5′-TGG​TCA​TCT​CCT​CAG​CAT​TGG​C-3′
*Hdac6*	Forward primer	5′-TCG​CTG​TCT​CAT​CCT​ACC​TGC​T-3′
​	Reverse primer	5′-GTC​AAA​GTT​GGC​ACC​TTC​ACG​G-3′
*Gapdh*	Forward primer	5′-CAT​CAC​TGC​CAC​CCA​GAA​GAC​TG-3′
​	Reverse primer	5′-ATG​CCA​GTG​AGC​TTC​CCG​TTC​AG-3′

### 2.10 Western blot analysis

Total protein from heart tissues and cells was extracted using RIPA lysis buffer (Thermo Fisher Scientific, Bremen, Germany). Samples were incubated on ice for 30 min and centrifuged at 4 °C for 15 min. Protein concentration was determined using a BCA assay kit (Epizyme Biotech, Shanghai, China). Equal amounts of protein were separated by 8%–12% SDS-PAGE (Epizyme Biotech, Shanghai, China) and transferred onto PVDF membranes (Millipore, Burlington, MA, United States). Membranes were blocked with 5% skim milk and incubated with primary antibodies overnight at 4 °C, followed by incubation with appropriate secondary antibodies for 1 h at room temperature. Protein bands were visualized using an enhanced chemiluminescence system. Quantitative analysis was performed using ImageJ software (NIH, United Statesa), and target protein expression levels were normalized to GAPDH.

### 2.11 AP-SMALDI-mass spectrometry imaging

Matrix application was performed using an ultrafine pneumatic sprayer (SMALDIPrep, TransMIT GmbH, Giessen, Germany). For positive-ion mode, 100 μL of DHB solution (30 mg/mL in acetone/H_2_O/TFA, 49.95:49.95:0.1, v/v/v) was applied at a flow rate of 10 μL/min with nitrogen pressure set to 1 bar. Mass spectrometry imaging (MSI) was performed using an Orbitrap Exploris 120 mass spectrometer (Thermo Fisher Scientific, Bremen, Germany) coupled with an AP-SMALDI5 AF ion source (TransMIT GmbH). The spatial resolution was set to 50 μm. Data acquisition and instrument control were performed using SMALDI Control software. Mass spectra were visualized using Xcalibur (Thermo Fisher Scientific), and data processing was conducted using MSiReader and Mirion software.

### 2.12 Measurement of carnitine content and calculation of acylcarnitines

The concentrations of free carnitine and total carnitine in H9c2 cells were determined using a commercial Free Carnitine and Total Carnitine Content Assay Kit (BC0675, Solarbio, Beijing, China) according to the manufacturer’s instructions and as previously described in published studies (Yi et al., 2026). Briefly, samples were homogenized in the extraction buffer and centrifuged to collect the supernatant. For total carnitine determination, a subset of the supernatant underwent alkaline hydrolysis to release conjugated carnitine before the neutralization step. The absolute contents of free and total carnitine were calculated based on the standard curve and normalized to the total protein concentration of each sample. Finally, the acylcarnitine content was calculated by subtracting the free carnitine concentration from the total carnitine concentration.

### 2.13 Determination of fatty acid oxidation (FAO) rate

The functional fatty acid oxidation (FAO) rate of H9c2 cardiomyocytes was evaluated utilizing an FAO Rate Assay Kit (BC0815, Solarbio, Beijing, China) following the manufacturer’s protocol and as previously described in published studies (Huynh et al., 2014). Cells from different treatment groups were washed with PBS, lysed using the specialized isolation buffer, and centrifuged to obtain the functional enzymatic matrix. The enzymatic reaction was initiated by adding the substrate mixture containing palmitoyl-CoA. The consumption of substrates or the concomitant generation of intermediates was monitored by tracking the absorbance dynamics at the specified wavelength using a spectrophotometer. The metabolic FAO rate was calculated and expressed as enzymatic units (U) per milligram of total protein, or further normalized relative to the Control group.

### 2.14 Metabolomics and lipidomics data analysis

Raw metabolomics and lipidomics data were processed using the corresponding vendor software for peak detection, alignment, and normalization. The resulting was processed and analyzed using MetaboAnalyst (version 4.0, www.metaboanalyst.ca). Prior to statistical analysis, data were normalized to a constant sum, log10 transformed, and autoscaled prior to multivariate analysis. Principal component analysis (PCA) was performed to performed to visualize clustering patterns and evaluate global metabolic alterations among groups. Differential metabolites and lipid species were identified using one-way analysis of variance (AVOVA) followed by Turkey’s *post hoc* test. To account for multiple testing and the high dimensionality of the omics datasets, p values were adjusted using the Benjamini–Hochberg procedure to control the false discovery rate (FDR). Metabolites and lipids were considered significantly altered when the FDR-adjusted p value (q value) ≤0.05 in combination with the designated fold-change threshold. Functional interpretation of significantly altered metabolites and lipids were performed using the Metabolite Set Enrichment Analysis (MSEA), MSEAlipid, and Pathway Analysis modules in MetaboAnalyst to identify significantly affected metabolic pathways and biological processes. The Benjamini–Hochberg FDR correction was consistently applied across all multi-omics datasets, including transcriptomics, lipidomics, and spatial metabolomics analyses.

### 2.15 Statistics analysis

All conventional data are presented as mean ± SEM unless otherwise stated. Sample sizes and statistical methods are indicated in the corresponding figure legends. For comparisons between two groups, an unpaired Student's *t*-test was used. For comparisons among multiple groups, a one-way analysis of variance (ANOVA) followed by Tukey’s *post hoc* test was performed.

## 3 Results

### 3.1 Transcriptomics reveals early metabolic disturbances in doxorubicin-induced cardiotoxicity

**FIGURE 1:**
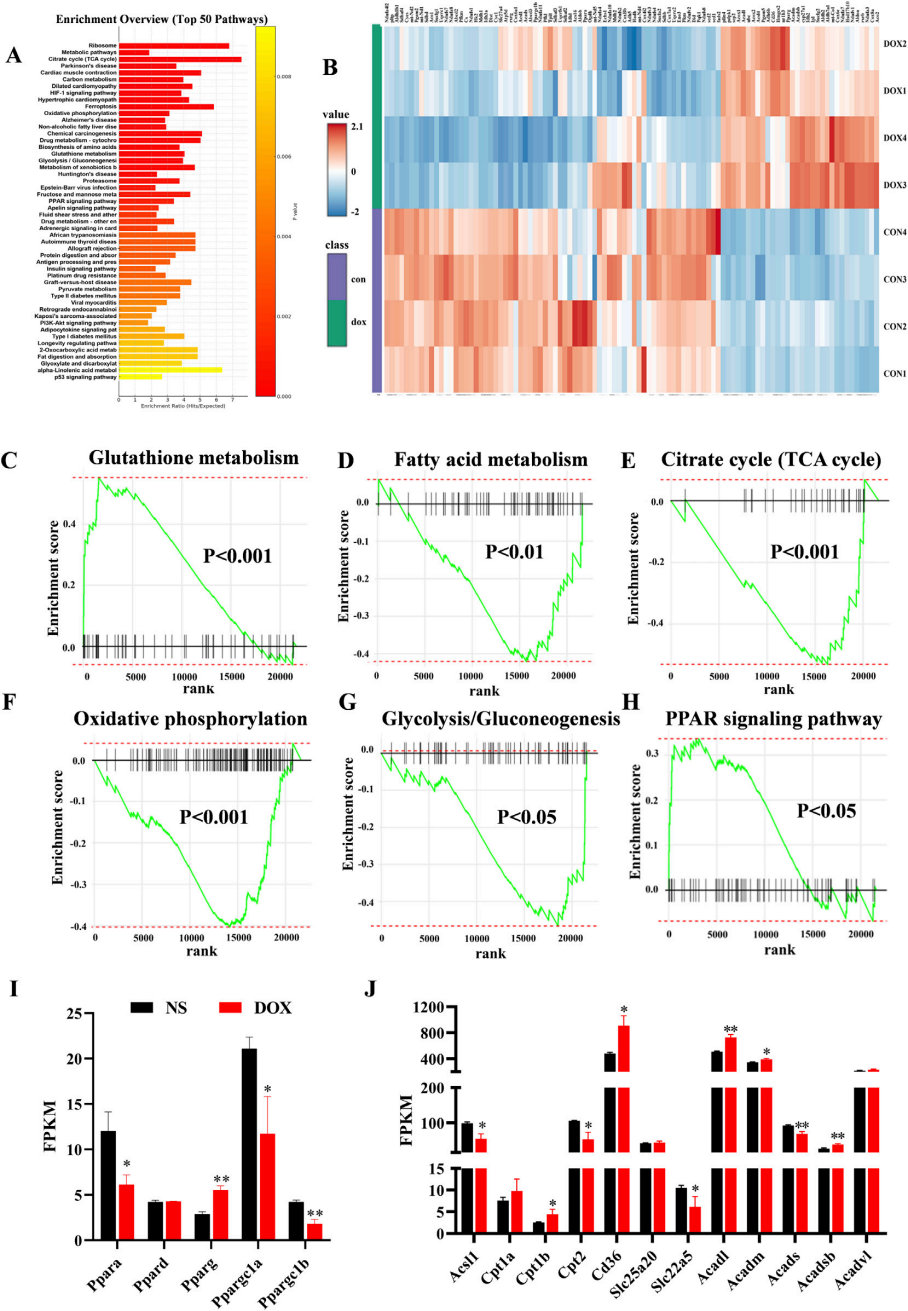
Transcriptomics reveals early metabolic disturbances in doxorubicin (Dox)-induced cardiotoxicity. **(A)** Kyoto Encyclopedia of Genes and Genomes (KEGG) enrichment analysis of DEGs; **(B)** Heatmap of DEGs associated with the PPAR signaling pathway, Fatty acid oxidation, Citrate cycle, Oxidative phosphorylation, Glycolysis/Gluconeogenesis in Dox-induced cardiac injury, based on data from GSE233644; **(C-H)** Results of Gene Set Enrichment Analysis (GSEA) of Glutathione metabolism **(C)**, fatty acid oxidation **(D)**, Citrate cycle **(E)**, Oxidative phosphorylation **(F)**, Glycolysis/Gluconeogenesis **(G)** and PPAR signaling pathway **(H)**. **(I)** Relative mRNA expression levels of PPARs (*Ppara*, *Ppard*, *Pparg*), *Ppargc1a* and *Ppargc1b* (upstream regulators of fatty acid metabolism) relative to controls; **(J)** Relative mRNA expression levels of genes associated with fatty acid oxidation. FPKM (fragments per kilobase of transcript per million fragments mapped reads) indicates the normalized RNA-seq expression values. Data are presented as mean ± SEM (n = 3). Statistical significance between two groups was determined using an unpaired Student's t-test. *P < 0.05, **P < 0.01, ***P < 0.001.

To investigate the early metabolic changes in acute cardiac injury, we analyzed dataset GSE233644 from the GEO database. In this model, mice received a single intraperitoneal injection of doxorubicin (15 mg/kg), and heart tissues were collected on day 5 for transcriptomic analysis. Pathway enrichment analysis of differentially expressed genes (DEGs) revealed significant enrichment of multiple metabolic pathways, including the tricarboxylic acid (TCA) cycle, oxidative phosphorylation, glutathione metabolism, glycolysis/gluconeogenesis, and fatty acid metabolism (Figure 1A). Heatmap analysis further confirmed marked transcriptional alterations in mitochondrial and electron transport chain genes, fatty acid metabolism, and the PPAR signaling pathway—transcription factors known to regulate cardiac metabolism (Figure 1B). Gene set enrichment analysis (GSEA) demonstrated that glutathione metabolism was significantly upregulated (Figure 1C), whereas most other metabolic pathways were downregulated (Figures 1D–G). As glutathione is a key intracellular antioxidant, this upregulation may reflect enhanced glutathione synthesis in response to oxidative stress. Interestingly, the PPAR signaling pathway was also significantly altered (Figure 1H). Among the three PPAR isoforms, *Ppara* (predominantly expressed in the heart) was significantly downregulated, *Ppard* showed no significant change, while *Pparg* (normally low in the heart) was significantly upregulated. The transcriptional coactivators PPARGC1A and PPARGC1B were both markedly downregulated (Figure 1I). In the analysis of genes related to fatty acid oxidation (FAO), we found that the expression of carnitine palmitoyltransferase 1B (*Cpt1b*)—the muscle-specific isoform responsible for converting activated long-chain acyl-CoA to acylcarnitine—was markedly upregulated, whereas carnitine palmitoyltransferase 2 (*Cpt2*), which reconverts acylcarnitine to long-chain acyl-CoA in the mitochondrial matrix, was significantly downregulated. In addition, the expression of *Cd36*, the plasma membrane transporter mediating long-chain fatty acid uptake, as well as Acyl-CoA Dehydrogenase Very Long Chain (*Acadvl*) and Acyl-CoA Dehydrogenase Medium Chain (*Acadm*), was also notably increased. In contrast, genes such as the Solute Carrier Family 22 Member 5 (*Slc22a5*), carnitine–acylcarnitine translocase (*Slc25a20*), Acyl-CoA Synthetase Long Chain Family Member 1 (*Acsl1*), and Acyl-CoA Dehydrogenase Short Chain (*Acads*) were all significantly downregulated (Figure 1J). In addition, mitochondrial DNA–encoded electron transport chain genes, as well as nuclear-encoded subunits of complexes I–IV, were significantly downregulated (Supplementary Figure S1). Collectively, these results indicate that acute cardiac injury induces profound metabolic disturbances, particularly in fatty acid oxidation, which may be driven by abnormal activation of PPARs.

### 3.2 Lipidomic profiling uncovers perturbed sphingolipid metabolism and acylcarnitine accumulation in doxorubicin-induced cardiotoxicity

**FIGURE 2:**
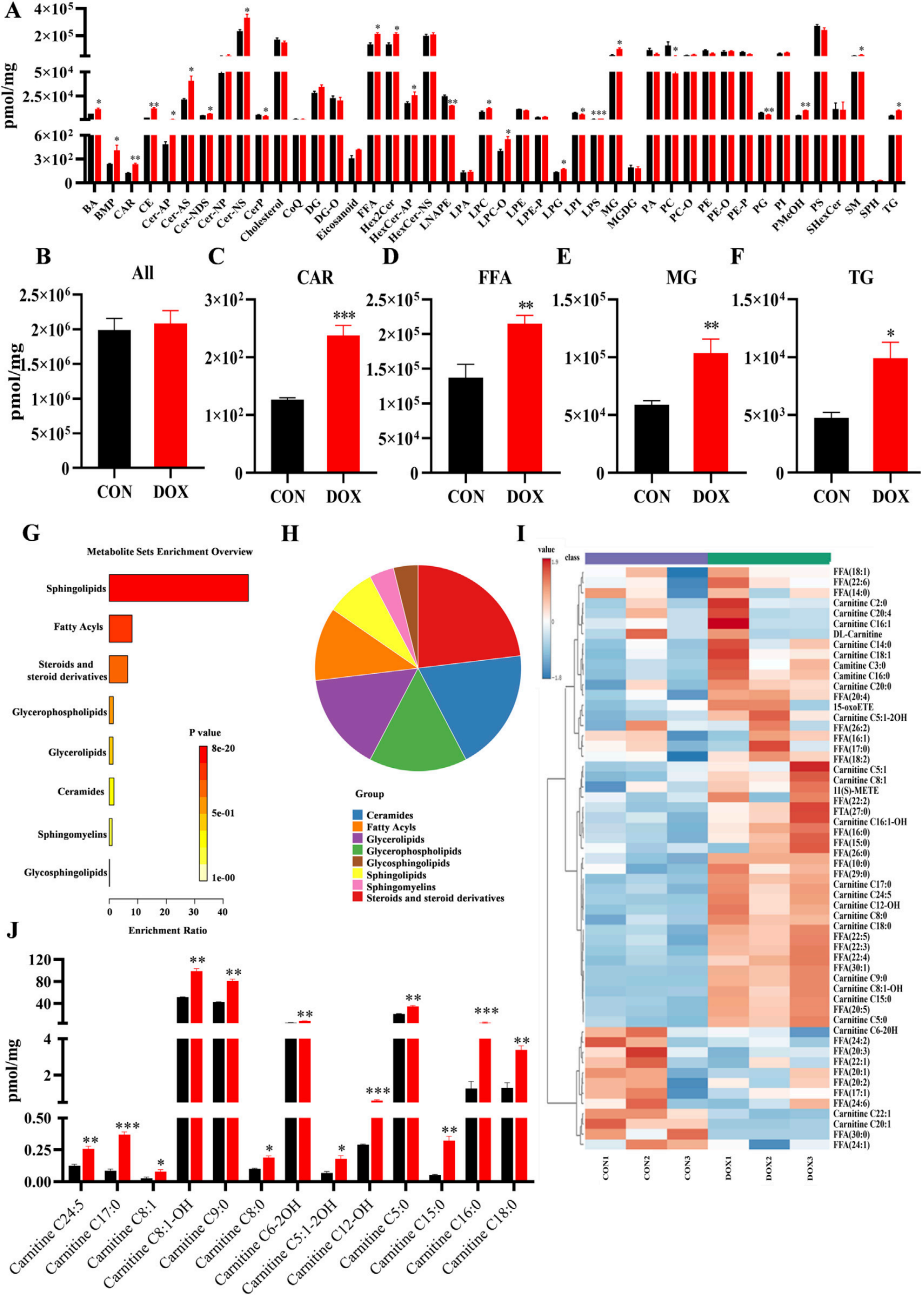
Lipidomic profiling uncovers perturbed sphingolipid metabolism and acylcarnitine accumulation in doxorubicin-induced cardiotoxicity. **(A)** Relative abundance of major lipid subclasses; **(B)** Changes in total lipid content; **(C-F)** Alterations in acylcarnitine (CAR, **(C)**, free fatty acid (FFA, **(D)**, monoacylglycerol (MG, **(E)** and diacylglycerol (DG, **(F)**. **(G)** Lipid set enrichment analysis (MSEA^Lipid^) of differentially abundant lipids in the DOX group compared with Con group. Significant enrichment was observed for Sphingolipid (P < 0.001) and fatty acyls (P < 0.001); Bonferroni-corrected P values were calculated using the QEA module of MetaboAnalyst based on the globaltest algorithm and a generalized linear model to estimate the Q-stat for each lipid set. **(H)** Composition of Lipid subclasses among differentially abundant lipids in DOX group, relative to Con group. Each color represents a distinct lipid subclass, and the area of each colored segment indicates its relative proportion. **(I)** Heatmap of differential abundant lipids (P < 0.05, n = 3, Student’s test). Colors represent normalized lipid signal intensities, highlighting lipids increased or decreased in the DOX group, relative to the Con group. **(J)** Relative abundance of acylcarnitines in DOX group, relative to Con group. Data are presented as mean ± SEM (n = 3). Statistical differences between the DOX and Con groups were analyzed using an unpaired Student's t-test. *P < 0.05, **P < 0.01, ***P < 0.001.

To examine lipid metabolic responses at the early stage of injury, we performed semi-quantitative lipidomic on H9C2 cardiomyocyte from control and doxorubicin-treated groups. A total of 44 lipid subclasses comprising 1,148 species were detected. Among these, 530 lipids were significantly altered, with 284 downregulated and 246 upregulated. Concentration analysis of the 44 subclasses revealed significant changes in 23, including 19 upregulated (e.g., BA, BMP, CAR, CE, Cer-AP, Cer-AS, Cer-NDS, Cer-NS, FFA, Hex2Cer, HexCer-AP, LPC, LPC-O, LPG, LPS, MG, PMeOH, SM, TG) and 4 downregulated (e.g., CerP, LNAPE, PC, PG) subclasses (Figure 2A). Notably, total lipid abundance did not differ significantly between groups, suggesting that DOX primarily induces lipid structural remodeling rather than altering overall lipid abundance (Figure 2B). Consistent with this, levels of several lipid metabolic intermediates, including carnitine (CAR), free fatty acids (FFA), monoacylglycerol (MG), and triglycerides (TG), were markedly increased in the DOX-treated group (Figures 2C–F).

Applying rigorous thresholds of an FDR-adjusted p-value (q-value) ≤ 0.05 and |log2FC| ≥ 1, enrichment analysis identified sphingolipids and fatty acyls as the most significantly affected subclasses (Figures 2G,H). The sphingolipid group included 14 altered species, such as Cer(d18:1/23:0), Cer(d18:1/16:0), Cer(d18:1/20:0), Cer(d18:1/22:0), Cer(d18:1/24:1), GlcCer(d18:1/24:1), GalCer(d18:1/14:0), SM(d18:2/24:1), and others. The fatty acyl group contained 17 altered species, including palmitic acid, palmitoylcarnitine, octanoylcarnitine, stearoylcarnitine, docosatrienoic acid, docosapentaenoic acid (22n-3), glutaconylcarnitine, and 3-hydroxydodecanoylcarnitine (Figure 2I). Ceramides are known to activate apoptotic pathways, inhibit pro-survival signaling, disrupt insulin signaling, impair glucose utilization, and exacerbate metabolic dysfunction, thereby contributing to cardiac injury (McCaffrey and Ibdah, 2025; Augusto et al., 2024; Park et al., 2022). Sphingolipid metabolites also regulate inflammation, and their dysregulation may promote myocardial inflammatory responses (York et al., 2024; Jiang et al., 2025; Gruevska et al., 2025; Phan et al., 2024; Foran et al., 2024). Consistent with this, most ceramide subclasses were significantly upregulated, indicating severe sphingolipid imbalance and activation of stress/apoptotic signaling. The heart relies heavily on fatty acid β-oxidation for energy supply; impaired oxidation can severely compromise cardiac function. Previous studies have shown that DOX impairs β-oxidation and ATP synthesis, leading to accumulation of metabolic intermediates. In line with this, we observed increased levels of multiple medium- and long-chain acylcarnitines, including C8:0, C8:1, C9:0, C15:0, C16:0, C17:0, and C18:0 (Figure 2J). Since acylcarnitines serve as carriers that transport fatty acids into mitochondria for β-oxidation, their accumulation reflects a blockade in fatty acid oxidation. Taken together, these findings suggest that DOX-induced myocardial injury may be associated with abnormal accumulation of ceramides and acylcarnitines, reflecting profound disturbances in sphingolipid metabolism and fatty acid oxidation.

### 3.3 Mass spectrometry imaging (MSI) reveals impairment in long-chain fatty acid oxidation in doxorubicin-induced cardiotoxicity

To delineate the spatial distribution patterns of metabolites at the tissue level during the early phase of doxorubicin-induced cardiotoxicity, we performed untargeted spatial metabolomic analysis on cardiac tissues at the early injury stage. After processing the data following previously established methods (Luo et al., 2023a), principal component analysis (PCA) of ion peaks zshowed tight clustering within both DOX and CON groups, with clear separation between them (Supplementary Figure S2A). Using statistical analysis packages in Metaboanalyst, Statistical analysis of all m/z features (FC > 1.5 or <0.6, P ≤ 0.05) identified 1,307 significantly altered peaks, of which 331 upregulated and 975 downregulated in the DOX group (Supplementary Figure S2B-D). Metabolite set enrichment analysis (MSEA) of significant altered metabolites showed that 4 pathways were significantly enriched in Con group, including oxidation of branched chain fatty acids (P < 0.01), fructose and mannose degradation (P < 0.05), inositol phosphate metabolism (P < 0.05) and beta oxidation of very long chain fatty acids (P < 0.05), and 10 pathways were significantly enriched in DOX group, including purine metabolism (P < 0.001), pyrimidine metabolism (P < 0.001), pentose phosphate pathway (P < 0.001), pentose and glucuronate interconversions (P < 0.001), arginine biosynthesis (P < 0.001), alanine, aspartate and glutamate metabolism (P < 0.01), histidine metabolism (P < 0.01), nitrogen metabolism (P < 0.05), glycerolipid metabolism (P < 0.05) and thiamine metabolism (P < 0.05) (Supplementary Figure S2E,F). These results were consistent with previous studies (Tan et al., 2023; Wan et al., 2025) regarding both transcriptomic and metabolomic data, confirming the reliability of the research methodology.

**FIGURE 3:**
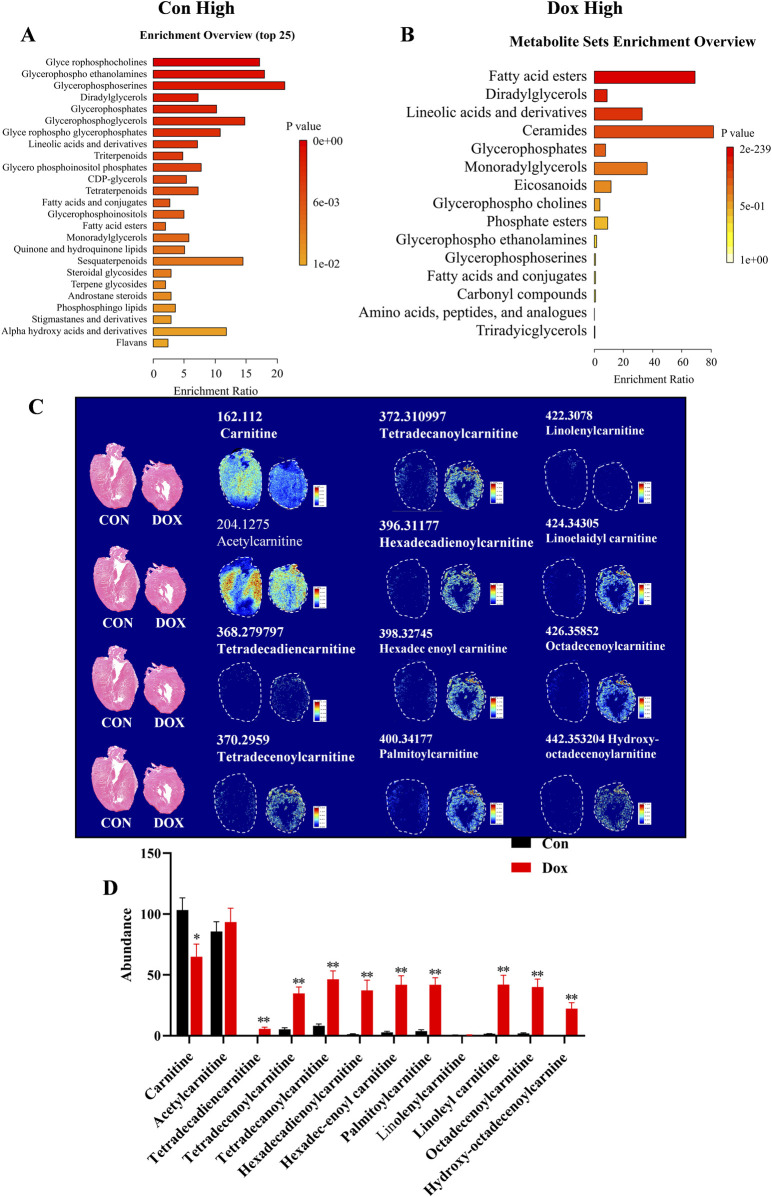
Mass spectrometry imaging analysis of metabolic patterns in doxorubicin (Dox)-induced cardiotoxicity. A,B. MSEA^lipid^ of differentially abundant lipids in DOX group and Con group based on MSI data. **(A)** MSEA^lipid^ of upregulated metabolites in CON group. **(B)** MSEA^lipid^ of upregulated metabolites in DOX group. **(C)** Hematoxylin and Eosin (HE) staining and representative MSI of spatial distributions of lipid metabolites in mice heart of CON and DOX group. **(D)** Relative abundance of carnitine, acetylcarnitine and acylcarnitines in DOX group, relative to Con group. Data are presented as mean ± SEM (n = 3). Statistical significance between two groups was evaluated using an unpaired Student's t-test. *P < 0.05, **P < 0.01, ***P < 0.001.

To broadly analyze the myriad lipid species enriched in DOX group, relative to Con, MSEAlipid was performed on the MSI data. MSEAlipid showed that enrichment of glycerophosphocholines, glycerophosphoethanolamines, glycerophosphoserines, diradylglycerols, glycerophosphates, glycerophosphoglycerols, glycerophosphoglycerophosphates, lineolic acids and derivatives et al. in CON hearts, whereas fatty acid esters, diradylglycerols, ceramides, monoradylglycerols were significant enriched in DOX group (Figures 3A,B), consistent with bulk lipidomic data. We next quantified carnitine derivatives, key biomarkers of mitochondrial substrate utilization as established in our prior work (Luo et al., 2023a; Luo et al., 2023b). Myocardial carnitine levels were significantly depleted, consistent with increased consumption or sequestration. While acetylcarnitine was modestly elevated, suggesting a compensatory shift toward glucose/pyruvate oxidation or preserved short-chain FAO, there was a pronounced accumulation of multiple long-chain acylcarnitines (C14–C18, including palmitoylcarnitine, linoleylcarnitine, octadecenoylcarnitine). This profile indicates a specific impairment in long-chain fatty acid oxidation, leading to metabolic intermediate accumulation and mitochondrial stress. Spatial analysis revealed no interventricular differences, supporting the notion of a global myocardial metabolic derangement (Figures 3C,D).

### 3.4 Network pharmacology analysis of potential regulatory targets of CGA in doxorubicin-induced cardiotoxicity

**FIGURE 4:**
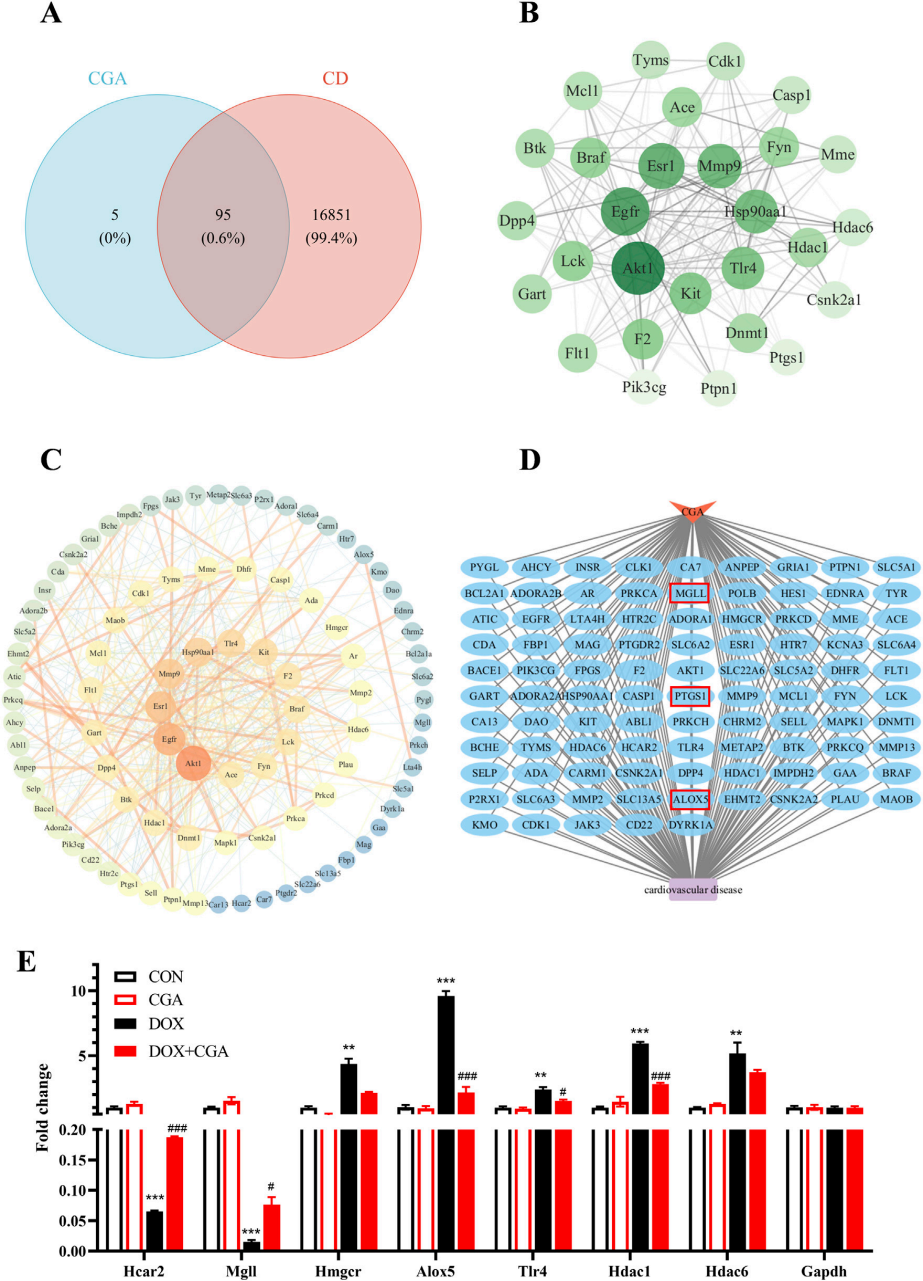
Overlaping target genes between CGA and Cardiovascular disease (CD) and the drug-compound-target-disease network. **(A)** Venn diagram of intersection targets of CGA and CD. Blue represents the targets of CGA and yellow represents the targets of CD. There were 98 intersecting targets. **(B)** Network analysis of shared core targets between CGA and CD; **(C)** PPI network of hub protein related to CAG and CD; **(D)** The integrated drug-target-disease network analysis of CGA in relation to CD was visualized by Cytoscape. **(E)** Relative mRNA expression levels of selected core hub targets in H9c2 cells across different treatments. Data are presented as mean ± SEM (n = 3). Statistical significance among multiple groups was analyzed using one-way ANOVA followed by Tukey’s *post hoc* test. *P < 0.05, P < 0.01, *P < 0.001 vs. Control; #P < 0.05, ##P < 0.01, ###P < 0.001 vs. DOX.

In the present work, a total of 100 targets of CGA were predicted based on the TCMSP database, PubChem and Swiss Target Prediction database. The target genes related to cardiovascular disease were searched in the GeneCards Database, DrugBank and OMIM Databse, which were taken the intersection with the 16,946 active targets. After the deletion and integration of false positive targets and repeat targets, 95 potential targets of CGA against CD were obtained. Protein-protein interaction (PPI) analysis was performed with STRING database to identify core targets, and a visualized herb-active compound-core target network was generated in Cytoscape to predict the relevant pathways (Figures 4A–D). Functional annotation of these predicted targets suggested that CGA may exert pleiotropic regulatory effects across multiple biological processes. The analysis identified potential CGA targets, including HCAR2, MGLL, and HMGCR, are involved in lipid catabolism and energy homeostasis, whereas, ALOX5, LTA4H, and TLR4 represent key nodes in canonical inflammatory signaling pathways, and their aberrant activation can lead to myocardial tissue injury. In addition, epigenetic regulators such as HDAC1, HDAC6, and CARM1 may modulate transcriptional programs associated with metabolic and inflammatory response (Figure 4D). These observations suggeste that CGA may act as a multi-target modulator capable of influencing interconnected metabolic and inflammatory networks in the cardiovascular system.

To provide experimental context for these *in silico* predictions, we evaluated the mRNA expression of a selected panel of core hub targets in H9c2 cells across different treatments. Consistent with the network predictions, DOX severely dysregulated key nodes across multiple pathways (Figure 4E). The expression levels of pro-inflammatory drivers (Alox5, Tlr4), the cholesterol synthesis enzyme (Hmgcr), and epigenetic repressors (Hdac1, Hdac6) were drastically upregulated by DOX. Conversely, targets essential for lipolysis and protective lipid homeostasis, such as Mgll (which hydrolyzes monoacylglycerols) and the receptor Hcar2, were profoundly suppressed. CGA treatment significantly reversed these pathological alterations, suggesting a general restoration of metabolic and inflammatory homeostasis.

Importantly, although these network-derived targets do not directly map onto the FAO–acylcarnitine axis identified in our multi-omics and spatial metabolomics analyses, they likely function as upstream modulators that converge on mitochondrial lipid metabolic remodeling. In this context, the FAO impairment and acylcarnitine accumulation represent the core metabolic phenotype, whereas the predicted CGA target network may contribute to its regulation through coordinated effects on inflammation, lipid handling, and transcriptional control. Together, these findings support a multi-layered regulatory model in which CGA modulates both upstream signaling networks and downstream metabolic substrate remodeling in DOX-induced cardiotoxicity.

### 3.5 CGA ameliorates DOX-induced cardiac dysfunction

**FIGURE 5:**
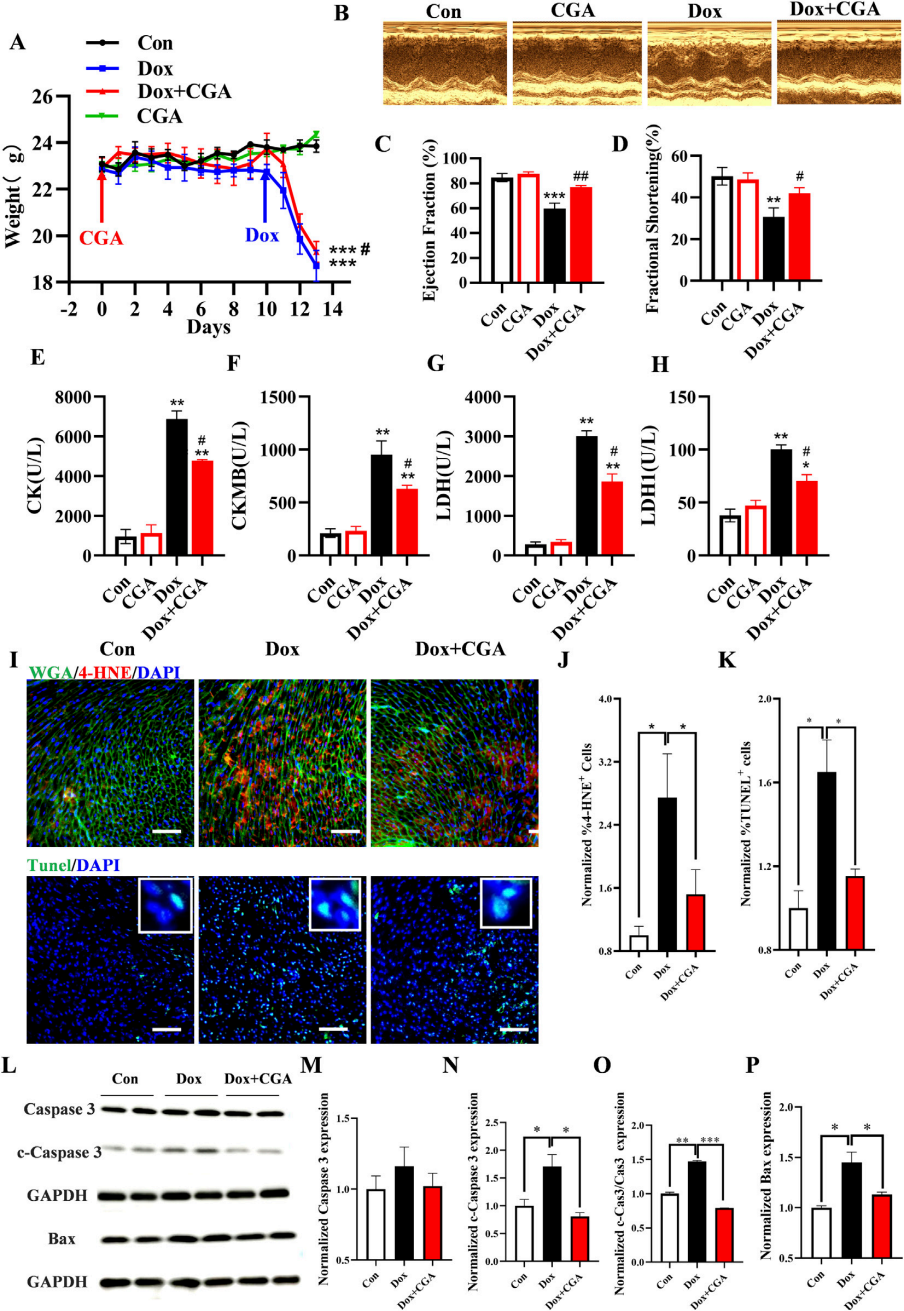
CGA attenuated DOX-induced cardiac injury in mice by reducing apoptosis and oxidative stress. **(A)** Changes in mice body weight. CGA or ddH2O was administered via oral gavage starting on day 0. Ten days later, a single intraperitoneal injection of doxorubicin (15 mg/kg) or saline was given. Body weight declined significantly after doxorubicin injection, whereas CGA attenuated the weight loss. **(B)** Representative M-mode echocardiography images of mice in different groups. **(C,D)** Echocardiographic evaluation of Left Ventricular Ejection Fraction **(C)** and Fractional Shortening **(D)**. **(E–H)** Serum levels of CK **(E)**, CKMB **(F)**, LDH **(G)**, and LDH1 **(H)**. **(I)** Representative fluorescence images of 4-HNE (upper panel) and TUNEL (lower panel) staining in cardiac tissues. **(J,K)** Quantification of the percentage of 4-HNE-positive cells **(J)** and TUNEL-positive cells **(K)**. **(L)** Western blot analysis of Caspase 3, c-Caspase 3, and Bax protein expression in cardiac tissues following doxorubicin injection, with or without oral CGA treatment. **(M–P)** Quantification of Caspase 3 **(M)**, cleaved Caspase 3 **(N)**, the ratio of cleaved Caspase-3 to total Caspase-3 **(O)**, and Bax **(P)** protein levels from replicate Western blots, shown relative to the vehicle control and normalized to GAPDH. Data are presented as mean ± SEM. * compared to Con group, *P < 0.05, P < 0.01, ***P < 0.001; # compared to Dox group, ^#^P < 0.05, ^##^P < 0.01, ^###^P < 0.001.

To investigate the potential role of CGA in doxorubicin-induced cardiotoxicity, we established a mouse model of acute doxorubicin-induced myocardial injury. Consistent with previous reports, doxorubicin-treated mice exhibited significant body weight loss and reduced survival rate compared to the control group (Figure 5A). Doxorubicin administration markedly elevated serum levels of creatine kinase (CK), creatine kinase MB (CK-MB), lactate dehydrogenase L (LDH-L) and lactate dehydrogenase 1 (LDH-1), accompanied by impaired cardiac function, as evidenced by reduced left ventricular ejection fraction and fractional shortening (Figures 5B–H). This doxorubicin-induced cardiac dysfunction likely contributed to the decreased survival. Importantly, the administration of CGA alone did not alter body weight, basal cardiac function, or myocardial enzyme levels, confirming its safety and lack of independent basal toxicity in healthy mice. Notably, CGA treatment partially or nearly completely abrogated these pathological alterations. The functional improvements were corroborated by histopathological analyses. Doxorubicin induced pronounced oxidative stress, as indicated by increased 4-hydroxynonenal (4-HNE) staining, and promoted cardiomyocyte apoptosis (Figures 5I–K), these effects were partially reversed by CGA treatment. As further confirmed by reduced expression of BAX and cleaved caspase-3, as well as cleaved to total caspase-3 ratio (Figures 5L–P). Collectively, these results indicate that CGA mitigates doxorubicin-induced oxidative stress and apoptosis *in vivo*.

### 3.6 CGA alleviated dox-induced oxidative stress by reduce acylcarnitine accumulation

**FIGURE 6:**
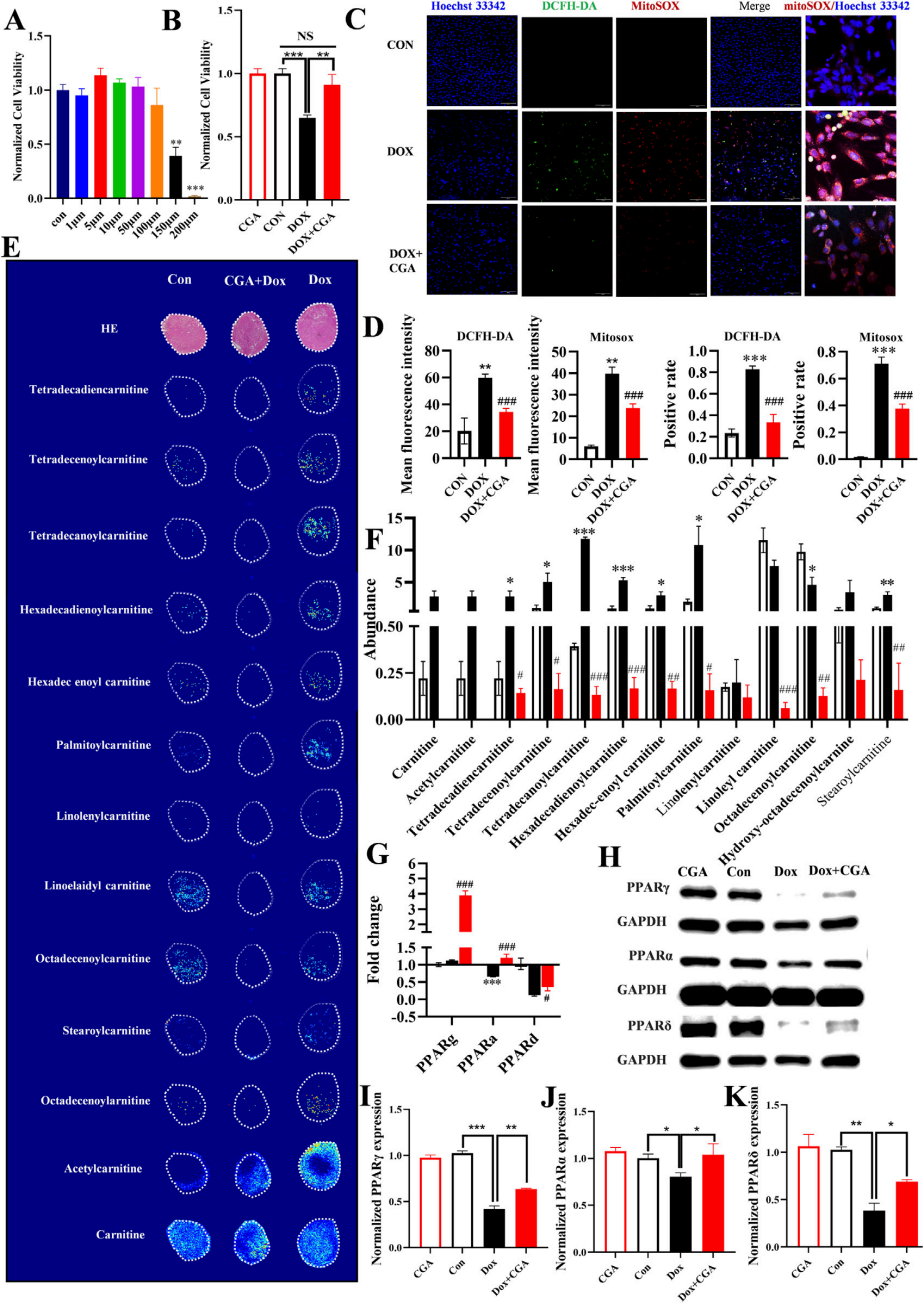
CGA reduced acylcarnitine accumulation and alleviated oxidative stress in doxorubicin-induced cardiac injury. **(A)** The effect of different concentration of CGA on cell viability. Low-dose CGA showed no significant cytotoxicity, while high-dose CGA impaired cell viability. **(B)** Treatment with 100 μM CGA significantly enhanced the viability of H9C2 cells exposed to doxorubicin. **(C)** Representative images of DCFH-DA and MitoSOX staining. DCFH-DA was used to assess overall cellular ROS, while MitoSOX specifically detected mitochondrial ROS. **(D)** Quantification of fluorescence intensity and positive cell percentage for DCFH-DA and MitoSOX staining. **(E)** MSI analysis showed that CGA reduced doxorubicin-induced acylcarnitine accumulation. Representative H&E staining and MSI images display the spatial distribution of acylcarnitines, carnitine and acetylcarnitine in mice heart. **(F)** Comparison of carnitine, acetylcarnitine, and acylcarnitine levels between DOX and CGA + DOX groups, expressed relative to the control group. **(G)** Relative mRNA expression levels of genes associated with PPARs. **(H)** Western bolt analysis of = PPARs protein expression in cardiac tissue following doxorubicin injection, with or without oral CGA treatment. **(I-K)** Quantification of PPARgamma **(I)**, PPARalpha 3 **(J)** and PPARdelta **(K)** protein levels from replicate Western blots, shown relative to the vehicle control and normalized to GAPDH. Data are presented as mean ± SEM. For *in vitro* experiments, n = 3; for *in vivo* experiments (e.g., MSI and tissue Western blots), n = 8. Statistical significance among multiple groups was calculated using one-way ANOVA followed by Tukey’s *post hoc* test. * compared to Con group, *P < 0.05, **P < 0.01, ***P < 0.001; # compared to Dox group. #P < 0.05, ##P < 0.01, ###P < 0.001.

To further confirm the protective effect of CGA against DOX-induced cardiomyocyte injury, we treated H9C2 cells with CGA in the presence or absence of DOX *in vitro*. Consistent with the *in vivo* findings, CGA at concentration 1–100 μM alone did not affect viability but significantly attenuated DOX-induced cytotoxicity (Figures 6A,B). ROS measurements showed that mitochondrial ROS was the dominant source of oxidative stress following doxorubicin treatment. Dichlorodihydrofluorescein diacetate (DCFH-DA) was used to measure total intracellular ROS, while MitoSOX, a mitochondria-targeted fluorogenic dye, was employed to detect mitochondrial ROS in live cells. DOX treatment elevated overall ROS levels, with MitoSOX staining indicating that mitochondria were the major source. Importantly, CGA reduced not only total ROS levels but also specifically mitigated mitochondrial ROS accumulation (Figures 6C,D).

To explore the mechanism underlying CGA-mediated suppression of mitochondria ROS, we performed MSI to assess acylcarnitine levels. Notably, CGA significantly alleviated the DOX-induced accumulation of acylcarnitines (Figures 6E,F). Since members of the PPAR family are critically involved in the pathogenesis of DOX-induced cardiomyopathy, and their dysregulation impairs fatty acid transport and β-oxidation, we next examined whether CGA exerts its cardioprotective effects through regulation of PPAR expression. Using qPCR and Western blot analyses of heart tissues from the DOX model, we found that DOX treatment markedly reduced the mRNA expression of PPARα and PPARδ, while having minimal effect on PPARγ. In contrast, CGA pretreatment significantly upregulated the transcription of PPARα, PPARδ, and PPARγ (Figure 6G). Western blot results further confirmed these findings: DOX downregulated protein expression of PPARα and PPARδ, whereas CGA pretreatment significantly increased protein levels of all three PPAR isoforms compared with the DOX group. Consistent with the *in vivo* functional observations, the CGA-only treatment did not autonomously alter the basal protein expression of PPARα, PPARγ, or PPARδ compared to the Control group, indicating its regulatory action is stress-responsive (Figures 6H–K). Collectively, these results suggest that CGA alleviates DOX-induced cardiac injury, at least in part, by restoring PPAR expression and thereby modulating the production of acylcarnitines, key intermediates of fatty acid oxidation, and mitochondrial ROS production.

### 3.7 Pharmacological validation of FAO-acylcarnitine axis in CGA-mediated cardioprotection

**FIGURE 7:**
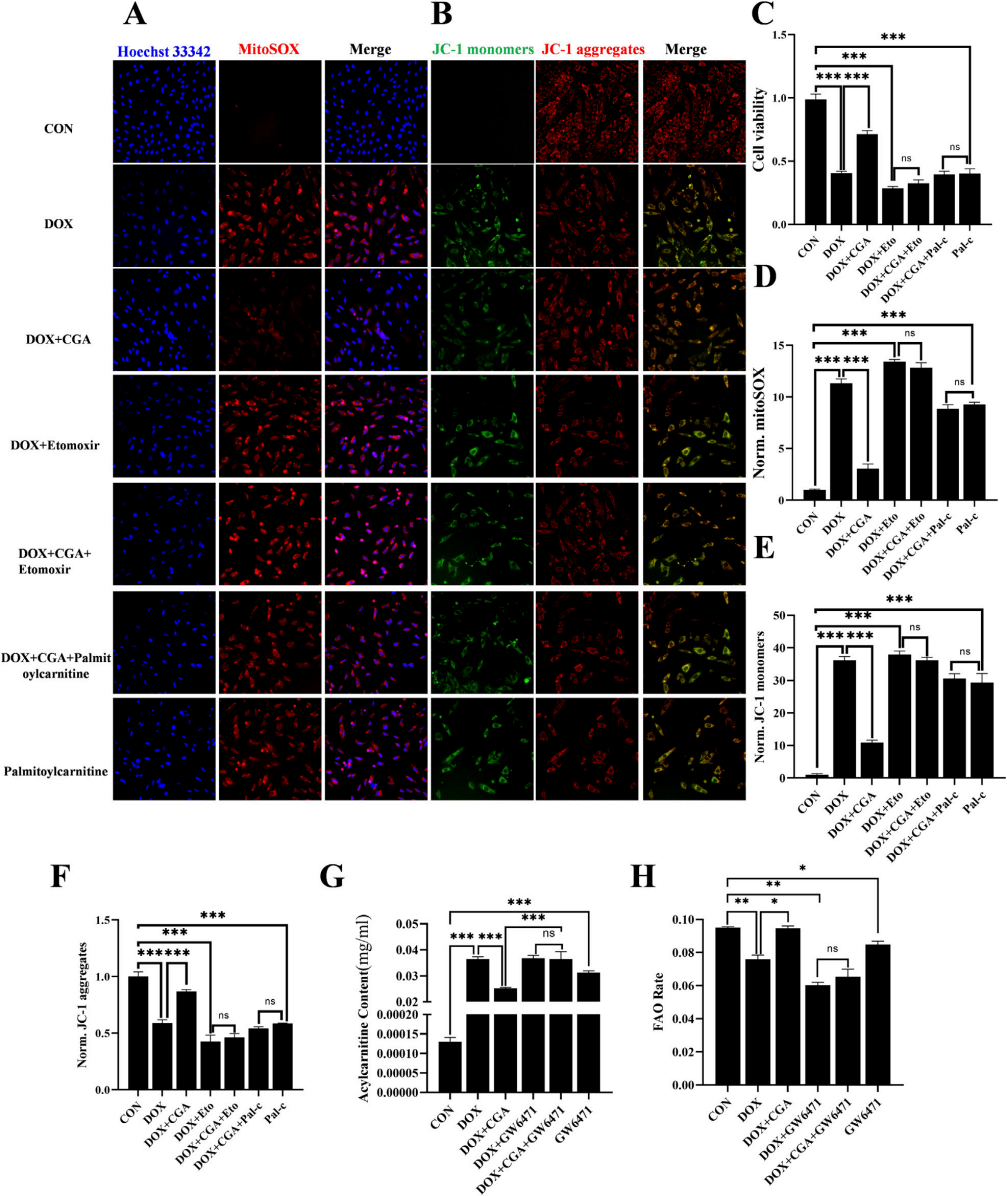
Pharmacological blockade of the PPARα-FAO axis and exogenous acylcarnitine accumulation abolish the cardioprotective and metabolic effects of CGA. **(A)** Representative images of mitochondrial ROS production detected by MitoSOX (red) and nuclei stained by Hoechst 33,342 (blue) in H9c2 cells. Cells were treated with DOX (5 μM), CGA (100 μM), the CPT1 inhibitor Etomoxir (Eto, 40 μM), and Palmitoylcarnitine (Pal-c, 25 μM) as indicated. **(B)** Representative fluorescence images of JC-1 staining for assessing mitochondrial membrane potential (ΔΨm). Red fluorescence represents JC-1 aggregates (normal ΔΨm), while green fluorescence indicates JC-1 monomers (depolarized ΔΨm). **(C)** Cell viability across different treatment groups determined by CCK-8 assay. **(D)** Quantification of normalized MitoSOX fluorescence intensity. **(E-F)** Quantification of normalized JC-1 monomers **(E)** and JC-1 aggregates **(F)**. **(G)** Absolute acylcarnitine content (mg/mL) in H9c2 cells under different treatment conditions including the specific PPARα antagonist GW6471. Acylcarnitine levels were calculated as the difference between total carnitine and free carnitine concentrations. **(H)** Direct measurement of the Fatty Acid Oxidation (FAO) rate in H9c2 cells. The restoration of FAO flux and the clearance of acylcarnitines by CGA are completely abolished when PPARα is inhibited by GW6471. Data are expressed as mean ± SEM(n = 3). Statistical differences among multiple groups were analyzed using one-way ANOVA followed by Tukey’s *post hoc* test. *P < 0.05, **P < 0.01, ***P < 0.001; ns indicates no significant difference between the specifically indicated groups.

To causally determine whether impared fatty acid oxidation (FAO) and acylcarnitine accumulation are causally involved in DIC and protective effect of CGA, we performed targeted pharmacological interventions in H9c2 cells. Exogenous palmitoylcarnitine (Pal-c, 25 μM) was used to mimic intracellular acylcarnitine accumulation, while etomoxir (Eto, 40 μM) and GW6471 were employed to inhibit FAO flux and PPARα activity respectively. Notably, Pal-c treatment alone significantly decreased cell viability (Figure 7C), provoked extensive mitochondrial ROS production (Figures 7A,D), and induced severe mitochondrial membrane depolarization, as evidenced by an increase in JC-1 monomers and a corresponding decrease in JC-1 aggregates (Figures 7B,E,F). These phenomena closely mimic the pathological phenotype of DOX. Furthermore, while CGA effectively protected against DOX-induced injury, the exogenous addition of Pal-c (DOX + CGA + Pal-c) completely abrogated the protective effects of CGA on cell viability and mitochondrial homeostasis (Figures 7A–F). Similarly, the blockade of CPT1 by Etomoxir (DOX + CGA + Eto) also nullified the therapeutic efficacy of CGA. These findings indicate that acylcarnitine accumulation is merely a metabolic byproduct but is sufficient to drive mitochondrial injury and lipotoxic cardiomyocyte death, and that CGA-mediated cardioprotection critically depends on maintaining physiological FAO flux and preventing acylcarnitine overload.

To further define the upstream regulatory mechanism, we assessed the role of PPARα signaling in FAO restoration. DOX markedly suppressed FAO activity and promoted acylcarnitine accumulation, whereas CGA effectively restored FAO flux and reduced lipid intermediate burden (Figures 7G,H). However, pharmacological inhibition of PPARα with GW6471 completely abolished these beneficial effects, preventing FAO recovery and leading to re-accumulation of acylcarnitines despite CGA treatment. Basal PPARα inhibition alone also disrupted lipid metabolic homeostasis. Collectively, these data demonstrate that activation of the PPARα–FAO axis is essential for CGA-driven metabolic remodeling and is required for the clearance of toxic acylcarnitines and the preservation of mitochondrial integrity.

## 4 Discussion

This study identifies impaired fatty acid oxidation (FAO) and acylcarnitine accumulation as key upstream drivers of doxorubicin-induced cardiotoxicity. Our findings extend the current paradigm that metabolic remodeling is merely a consequence of mitochondrial dysfunction and instead support a pathogenic role for disrupted metabolic substrate utilization in the early stages of cardiac injury. Mechanistically, chlorogenic acid restored PPAR-dependent FAO, reduced acylcarnitine burden, and alleviated DOX-induced oxidative stress, apoptosis, and cardiac dysfunction. These results highlight metabolic flux restoration as a promising therapeutic approach for preventing anthracycline-associated cardiotoxicity.

Preclinical models of DIC generally employ either a single high-dose DOX injection to induce acute cardiac injury or repeated low-dose administration to model cumulative cardiotoxicity (Xiao et al., 2023; Wa et al., 2023; Hu et al., 2019; Kuno et al., 2023). While chronic models better recapitulate the progressive cardiac dysfunction and structural remodeling observed in cancer survivors, the primary objective of the present study was to identify the early metabolic disturbances that initiate DOX-induced cardiotoxicity (He et al., 2026). Therefore, we employed an acute DOX exposure model, which is widely used to capture proximal molecular and metabolic responses before the onset of overt pathological remodeling. Transcriptomic profiling revealed extensive metabolic perturbations shortly after DOX exposure. In addition to the expected suppression of oxidative phosphorylation and TCA cycle pathways, fatty acid metabolism emerged as one of the most significantly affected processes. Notably, the expression of PPARα and its transcriptional coactivators PGC-1α and PGC-1β was markedly reduced, suggesting early impairment of the transcriptional program governing cardiac fatty acid utilization. Consistent with these findings, genes involved in mitochondrial fatty acid transport and β-oxidation were significantly downregulated. Meanwhile, activation of the glutathione metabolism pathway indicated that cardiomyocytes had already entered a metabolically stressed state in response to DOX exposure.

Lipidomic analyses further demonstrated that DOX rapidly induced the accumulation of multiple medium- and long-chain acylcarnitines together with ceramides in cardiomyocytes. Because acylcarnitines serve as essential intermediates in mitochondrial fatty acid transport, their accumulation is widely regarded as a functional indicator of impaired fatty acid β-oxidation. Spatial metabolomics confirmed widespread *in situ* accumulation of long-chain acylcarnitines accompanied by depletion of L-carnitine and acetylcarnitine, supporting the presence of an active metabolic bottleneck in fatty acid utilization. Importantly, mechanistic experiments further supported a causal role for disruption of the PPARα–FAO axis. Pharmacological inhibition of PPARα largely abolished the protective effects of CGA on fatty acid oxidation and acylcarnitine accumulation, indicating that restoration of PPARα signaling is required for the metabolic benefits of CGA. Moreover, exogenous palmitoylcarnitine treatment attenuated the cardioprotective effects of CGA, suggesting that acylcarnitine accumulation is not merely a biomarker of metabolic dysfunction but may actively contribute to cardiomyocyte injury. Collectively, these findings identify impaired PPARα-dependent fatty acid oxidation and subsequent acylcarnitine accumulation as early metabolic events in DOX-induced cardiotoxicity. Although the present study was not designed to investigate the long-term progression of cardiotoxicity, future studies employing chronic DOX exposure models and human-relevant cardiomyocyte systems will be valuable for determining whether these early metabolic abnormalities persist during later stages of anthracycline-induced cardiac injury.

Chlorogenic acid (CGA) has been widely reported to exert cardioprotective effects in multiple experimental models of cardiovascular injury, including myocardial ischemia rnduced cardiomyocyte damage, cardiac hypertrophy, and fibrosis (Li et al., 2020; He et al., 2024; Ling et al., 2025). In the present study, CGA effectively restored PPAR-dependent fatty acid oxidation, reduced acylcarnitine accumulation, and attenuated DOX-induced cardiac dysfunction, further supporting its therapeutic potential as a metabolic modulator in anthracycline cardiotoxicity.

From a translational perspective, the dosage employed in this study falls within a clinically relevant range. Based on body surface area normalization, the mouse dose of 100 mg/kg/day corresponds to a human equivalent dose of approximately 8.1 mg/kg/day, or about 480 mg/day for a 60-kg adult. Notably, clinical studies investigating CGA supplementation for metabolic and cardiovascular disorders have commonly used oral doses ranging from 140 to 1200 mg/day with favorable tolerability (Yu et al., 2022; Huang et al., 2025). In addition, Amano et al. (2019) evaluated the safety profile of CGA and its major metabolites using *in vitro* and *ex vivo* profiling assays according to the ICH S7A guideline and reported no significant safety concerns, supporting the potential utility of CGA as a therapeutic agent.

Nevertheless, the translational application of CGA remains influenced by its pharmacokinetic characteristics. Although accumulating evidence supports beneficial cardiovascular and metabolic effects, its absorption and bioavailability remain incompletely understood and are subject to considerable interindividual variability. Differences in gastrointestinal absorption, microbial metabolism, hepatic biotransformation, and excretion contribute to substantial variability among both preclinical and clinical studies. Current evidence suggests that CGA can be absorbed through at least two distinct routes: rapid absorption in the stomach and/or upper gastrointestinal tract, and slower absorption of intact CGA throughout the small intestine (Lu et al., 2020). Consistent with this concept, Mubarak et al. reported that acute ingestion of 400 mg pure CGA (approximately equivalent to that contained in two cups of coffee) resulted in measurable plasma concentrations of intact CGA within 2.5 h in healthy volunteers (Mubarak et al., 2012). Similarly, Lafay et al. detected CGA in rat plasma as early as 1.5 h after dietary administration, indicating relatively rapid systemic uptake (Lafay et al., 2006). Despite these observations, the absolute bioavailability of CGA remains controversial because extensive metabolism occurs before and after absorption.

Another important translational consideration is whether CGA might compromise the antitumor efficacy of DOX. Although this question was not directly addressed in the current study, available evidence does not suggest an antagonistic interaction between CGA and DOX. On the contrary, previous studies have demonstrated that CGA possesses intrinsic antitumor and immunomodulatory activities, including suppression of glioma progression through regulation of tumor-associated macrophage polarization (Xue et al., 2017; Kang et al., 2023). Furthermore, a recent study reported that co-delivery of CGA and DOX significantly enhanced antitumor efficacy in a melanoma model, suggesting that CGA may be compatible with, and potentially augment, the anticancer activity of DOX (Zhu et al., 2024). Nevertheless, these findings cannot substitute for direct evaluation in tumor-bearing models of DOX-induced cardiotoxicity, where both cardioprotective efficacy and tumor suppression can be assessed simultaneously. Therefore, the current findings should be interpreted primarily as mechanistic proof-of-concept demonstrating that pharmacological modulation of the PPAR–FAO–acylcarnitine axis can mitigate DOX-induced cardiotoxicity. Further pharmacokinetic studies and clinically relevant dosing investigations will be required to determine whether sufficient concentrations of CGA or its active metabolites can be achieved and maintained in humans to reproduce the cardioprotective effects observed in experimental models.

In terms of cardioprotection, CGA exerts remarkable protective effects on the heart. It can stabilize mitochondrial and lysosomal functions to alleviate isoproterenol-induced myocardial injury (Akila et al., 2017); inhibit the NF-κB and JNK signaling pathways to protect cardiomyocytes from TNF-α-induced damage (Tian et al., 2019); and attenuate H_2_O_2_-induced cardiomyocyte apoptosis (Yu et al., 2016). In animal models, CGA mitigates myocardial ischemia–reperfusion injury in mice by inhibiting Lnc Neat1/NLRP3 inflammasome–mediated pyroptosis (Chai et al., 2023). CGA also exhibits potential in suppressing cardiac hypertrophy and fibrosis through multiple mechanisms, including inhibition of the Wnt/β-catenin signaling pathway (He et al., 2024), activation of the NO/cGMP/PKG pathway (Qin et al., 2021), upregulation of sphingosine-1-phosphate receptor 1 to reduce endoplasmic reticulum stress (Ping et al., 2024), and downregulation of galectin-3 expression (Kim et al., 2025).

In doxorubicin (DOX)–induced cardiotoxicity models, CGA similarly displays potent cardioprotective activity. It activates the Nrf2/HO-1 pathway and modulates kynurenine signaling to exert anti-apoptotic effects (Cicek et al., 2023). Consistent with these findings, our study further demonstrates that CGA alleviates DOX-induced acute cardiac injury by reducing the excessive accumulation of lipid metabolic intermediates, particularly acylcarnitines. This mechanism aligns with previous reports highlighting the regulatory role of CGA in maintaining glucose and lipid metabolic homeostasis. Numerous studies have confirmed that CGA ameliorates obesity, diabetes, and related metabolic syndromes through modulation of key signaling molecules such as AMPK and ERK1/2. Regarding lipid metabolism, CGA has been shown to inhibit lipase activity and suppress the functions of several lipid metabolic enzymes—including fatty acid synthase, HMG-CoA reductase, and acyl-CoA:cholesterol acyltransferase—in high-fat diet (HFD)–fed mice. Simultaneously, CGA upregulates AMPK and CPT1 while inhibiting ACC activity, thereby reducing triglyceride and free fatty acid levels in the liver and plasma of HFD rats. Multiple studies have further revealed the key role of CGA in promoting fatty acid β-oxidation (Bruckbauer and Zemel, 2014). For instance, in oleic acid–induced HepG2 cells, CGA activates the AMPK/ACC/CPT-1 pathway to ameliorate hepatic steatosis, reduce lipid droplet accumulation and lipid content, enhance fatty acid β-oxidation, lower transaminase levels, and inhibit apoptosis (Ma et al., 2023). In a chronic endotoxin–induced rat model, CGA significantly improved hepatic lipid metabolic disorders by modulating fatty acid–metabolizing enzymes and activating AMPK, thereby decreasing serum triglyceride, free fatty acid, and hepatic cholesterol levels (Zhou et al., 2016). Moreover, CGA promotes human embryonic stem cell proliferation and lipid synthesis by enhancing fatty acid β-oxidation–derived acetyl-CoA production (Zong et al., 2025).

In summary, doxorubicin-induced metabolic disturbances, particularly alterations in lipid metabolism, may contribute to the early development of cardiac injury. The abnormal accumulation of acylcarnitines, key intermediates of fatty acid metabolism, was closely associated with DOX-induced acute cardiotoxicity and may reflect impaired fatty acid oxidation at an early stage. CGA attenuated these metabolic abnormalities, at least in part, through activation of the PPARs–FAO axis, thereby promoting fatty acid oxidation, improving metabolic homeostasis, and alleviating DOX-induced acute cardiac injury.

## Data Availability

The datasets presented in this study can be found in online repositories and the article’s Supplementary Material. The transcriptomic dataset analyzed in this study is available in the GEO database under accession number GSE233644 (https://www.ncbi.nlm.nih.gov/geo/query/acc.cgi?acc=GSE233644). The quantitative data for lipidomics and spatial metabolomics supporting the conclusions of this article are included directly in the Supplementary Material files. Further inquiries can be directed to the corresponding authors.

## Supplementary material

Supplementary Figure S1 Impaired Electron transport chain (ETC.) Gene Expression in Dox-Induced cardiomyopathy. **(A)** Mitochondrial DNA-encoded core genes of electron transport chain are significantly downregulated in Dox-induced cardiac injury. **(B)** Relative mRNA expression levels of genes in electron transport chain complexes I-IV compared to controls. FPKM indicates fragments per kilobase of transcript per million fragments mapped in RNA-seq. Data are expressed as mean ± SEM (n = 3). Statistical significance was determined using an unpaired Student's t-test. *P < 0.05, **P < 0.01, ***P < 0.001.

Supplementary Figure S2 Mass Spectrometry imaging analysis of DOX-induced cardiotoxicity. **(A)** Principal component analysis (PCA) shows the distributions of Con and DOX group; **(B)** Volcano plot of the analyzed dataset depicting top differentiat metabolites based on log2 fold change and adjusted p values. C.D, Metabolite set enrichment analysis (MSEA) of differentially abundant metabolites in Con and DOX group based on MSI data. **(C)** MSEA of significantly upregulated metabolites in Con group. Oxidation of branched chain fatty acids (P < 0.01), fructose and mannose degradation (P < 0.05), inositol phosphate metabolism (P < 0.05) and beta oxidation of very long chain fatty acids (P < 0.05); **(D)** MSEA of significantly upregulated metabolites in DOX group. Purine metabolism (P < 0.001), pyrimidine metabolism (P < 0.001), pentose phosphate pathway (P < 0.001), pentose and glucuronate interconversions (P < 0.001), arginine biosynthesis (P < 0.001), alanine, aspartate and glutamate metabolism (P < 0.01), histidine metabolism (P < 0.01), nitrogen metabolism (P < 0.05), glycerolipid metabolism (P < 0.05) and thiamine metabolism (P < 0.05); Bonferroni-corrected P values were calculated using the Quantitative Enrichment Analysis (QEA) module of MetaboAnalyst MSEA based on the globaltest algorithm and a generalized linear model to estimate the Q-stat for each metabolite set. Data are expressed as mean ± SEM (n = 3). Statistical significance for differential metabolites was calculated using an unpaired Student's t-test with FDR correction.

## Glossary

4-HNE: 4-Hydroxynonenal
ACC: Acetyl-CoA Carboxylase
AMPK: Adenosine Monophosphate-Activated Protein Kinase
AP-SMALDI: Atmospheric Pressure Scanning Microprobe Matrix-Assisted Laser Desorption/Ionization
BCA: Bicinchoninic Acid
CCK8: Cell Counting Kit-8
CGA: Chlorogenic Acid
CK: Creatine Kinase
CK-MB: Creatine Kinase-MB Isoenzyme
CPT1: Carnitine Palmitoyltransferase 1
DCFH-DA: Dichlorodihydrofluorescein Diacetate
DEGs: Differentially Expressed Genes
DHB: 2,5-Dihydroxybenzoic Acid
DIC: Doxorubicin-Induced Cardiotoxicity
DOX: Doxorubicin
FAO: Fatty Acid Oxidation
GSEA: Gene Set Enrichment Analysis
H&E: Hematoxylin and Eosin
LDH: Lactate Dehydrogenase
LVEF: Left Ventricular Ejection Fraction
LVFS: Left Ventricular Fractional Shortening
LVIDd: Left Ventricular Internal Diameter at End-Diastole
LVIDs: Left Ventricular Internal Diameter at End-Systole
MSI: Mass Spectrometry Imaging
MSEA: Metabolite Set Enrichment Analysis
PCA: Principal Component Analysis
PPAR: Peroxisome Proliferator-Activated Receptor
ROS: Reactive Oxygen Species
TCA: Tricarboxylic Acid (Cycle)
TFA: Trifluoroacetic Acid
